# A Third Biodiversity Metric in the Third Pole

**DOI:** 10.1111/gcb.70192

**Published:** 2025-04-12

**Authors:** Kenneth Oberlander

**Affiliations:** ^1^ H.G.W.J. Schweickerdt Herbarium, Department of Plant and Soil Sciences University of Pretoria Pretoria South Africa

**Keywords:** conservation, genetic diversity, protected areas, third pole

The readers of this journal need no introduction to the threat posed to biodiversity by anthropogenic factors such as habitat degradation and climate change. The (sometimes considerable) efforts by many national governments to increase biodiversity protection over the last few decades, via the establishment of protected areas for conservation, have nevertheless met with criticism, particularly with regard to the evidence base used for the establishment and expansion of such protected areas (Maxwell et al. 2020). This is particularly acute for regional planning efforts involving multiple governments, where the effects of national borders on biodiversity conservation may have profound consequences in the near future (Li et al. 2025). Ideally, such decisions should be made taking into account evidence from multiple different levels of biological organisation, but this is seldom achieved in reality.

Much of the diversity information utilised for conservation planning is at the species level, that is, simple metrics of species diversity or endemism. Alternatively, metrics above the species level are used, such as phylogenetic diversity (representing the amount of independent evolutionary history represented in a region) or functional or ecosystem diversity (maximising trait or ecosystem‐level diversity; Cadotte and Tucker 2018). In either case, the basic building blocks of what is conserved by such methods are species—treated as atomic, indivisible units. While undeniably valuable, these approaches can overlook the extraordinary wealth of readily available data, suggesting that members of a species are not all the same.

In the paper by Wambulwa et al. (2025), the authors set out to assess this third, somewhat overlooked biodiversity metric—genetic diversity, that is, diversity *below* the species level—as a potentially useful factor to include in evidence‐based conservation planning. While assessment of genetic structure and diversity underpins multiple fields of science, it is surprising how seldom it has been used—particularly in aggregate across large numbers of species—to help plan and expand protected areas. The implications of treating species as non‐atomic units—with interpopulational variability that is worth conserving and which may impact conservation success under scenarios of global change—have often been neglected in favour of other biodiversity metrics when it comes to protected area planning, particularly at the regional level. There is increased recognition that genetic diversity should play a greater role in future decision‐making around protected areas in general (Hoban et al. 2020).

Wambulwa et al. (2025) used genetic diversity patterns for this purpose in the Third Pole, a region corresponding to the Tibetan Plateau and associated high‐altitude mountain ranges of central Asia (Liu et al. 2022). The name is apt—outside the Arctic and Antarctic, this is the most ice‐rich region on Earth. The Third Pole is a prime candidate for this study—it hosts substantial plant diversity and endemism, has been the subject of focussed research on species response to climate change and has a network of protected areas across multiple political jurisdictions covering one‐third of its land area. More worryingly, at least some research on the Third Pole has indicated that the fragile ecosystems contained in this region are nearing collapse due to multiple anthropogenic factors (Liu et al. 2018), making planning for conservation urgent.

Wambulwa et al. (2025) started off by quantifying the patterns of genetic diversity for nearly 100 plant species for which genetic data were available across the Third Pole. There were clear latitudinal and longitudinal trends, with genetic diversity highest in the southeast. Notably, however, the authors found only a weak relationship between species and genetic diversity, suggesting that different aspects of diversity are being captured by each metric.

Determining potential predictors showed that topographic and climatic features were far more important than anthropogenic variables in explaining the observed genetic patterns, no matter the genetic marker system used. While their relative importance, and which specific climate and topographic variable contributed, did vary between different marker systems (possibly due to their different inheritance and dispersal mechanisms), the relatively small contribution of anthropogenic factors suggests that the patterns observed are mostly environmentally induced, have been minimally disturbed by humans, and that environmental variables may therefore be reliable predictors of future distributional changes.

Using environmental niche modelling, the authors then modelled potential distributions across all sampled species under present‐day conditions, under environmental conditions of the Last Glacial Maximum, and under two projected scenarios of future climate change. The last scenarios also allowed projections of how much potential in situ genetic diversity will be lost due to distributional change as the Third Pole warms. Significantly, these estimates included modelling of natural dispersal, so taking into effect possible migration as an option for preserving local genetic diversity.

Under future scenarios of climate change, species ranges are predicted to shift north by just over 40 km, and upslope by 40–80 m. While these changes may seem modest in absolute terms, they have a disproportionately sobering effect on predicted genetic diversity loss: approximately 7%–10% of total genetic diversity across the region, depending on the genetic marker system and future climate model that is used. It is even more alarming when considering population‐unique (private) diversity—predicted losses vary between 9% and 15%.

These are all important and worrying findings. However, where this study really adds to our knowledge is twofold. First, as evident in their data, species‐level and genetic diversity patterns do differ—they record different aspects of biodiversity. As a consequence, incorporating genetic diversity into future planning can lead to substantially different conservation foci: Over 70% of priority conservation areas newly identified by Wambulwa et al. (2025) lie outside the formal protected network in the Third Pole.

There are caveats to the study. Perhaps the most serious is that sampling effort is not concentrated equally across the Third Pole, with the bulk of sampled species data coming from the southeast (possibly concerning, given that this happens to be the locus of genetic diversity found in this study). This is unavoidable given the authors decision to use already published and publicly available data. It would be illuminating to include more species from the western portion of the range, which may reflect different biogeographic (and genetic) histories. The species sampling, while phylogenetically representative, is still sparse—less than 100 of an estimated 18,000 plant species in the Third Pole. That said, even the low species sampling makes a cogent case for considering genetic diversity.

To summarise, significant and interesting outputs from the Wambulwa et al. (2025) study are the overarching patterns of genetic diversity (across almost 100 species spanning the vascular plant tree of life) across the Third Pole, what factors appear to be driving these patterns (both currently and in the future), and how much genetic diversity might be lost under future climate change scenarios without direct human intervention. However, the real novelty value of this study is in how incorporating genetic diversity information changes planning scenarios for future protected areas. It is to be hoped that future such planning efforts take this message to heart.

## Author Contributions

**Kenneth Oberlander:** writing – original draft, writing – review and editing.

## Conflicts of Interest

The author declares no conflicts of interest.

## Data Availability

Data sharing not applicable to this article as no datasets were generated or analysed for the current article.
